# Supplementary material for: Relationship between the strength of craving as assessed by the Tobacco Craving Index and success of quitting smoking in Japanese smoking cessation therapy
Source: PLoS One. 2020 Dec 7;15(12):e0243374. doi: 10.1371/journal.pone.0243374 (PMC7721195; doi:10.1371/journal.pone.0243374)
Supplement: S1 Fig — (DOCX) [file pone.0243374.s001.docx]

**S1 Fig.**

**Tobacco Craving Index (TCI)**

| Level of craving | 0: I have no craving for smoking at all  1: I miss having something in my mouth  2: I can manage the craving  3: I keep having craving for smoking |
| --- | --- |
| Frequency of daily craving | 0: not at all 　 1: less than once a day  2: 1~3 times a day 3: 4 times or more a day |

**The patient was asked to rate their level of craving on a scale of 0-3 and frequency of daily craving on a scale of 0-3. The TCI grade (G) was determined by the patient’s ratings on the two questionnaire items according to the following chart. The TCI grade (G) ranges from 0 to 3.**

**TCI Grade**

**Level of craving**

**Strength of craving**

**Frequency of daily craving**

|  | **0** | **1** | **2** | **3** |
| --- | --- | --- | --- | --- |
| **0** | **G0** |  |  |  |
| **1** |  | **G1** | **G1** | **G2** |
| **2** |  | **G1** | **G2** | **G3** |
| **3** |  | **G1** | **G2** | **G3** |
